# Facile Fabrication of Z-Scheme Bi_2_WO_6_/WO_3_ Composites for Efficient Photodegradation of Bisphenol A with Peroxymonosulfate Activation

**DOI:** 10.3390/nano10040724

**Published:** 2020-04-11

**Authors:** Yongkui Huang, Shuangwu Kou, Xiaoting Zhang, Lei Wang, Peili Lu, Daijun Zhang

**Affiliations:** State Key Laboratory of Coal Mine Disaster Dynamics and Control, College of Environment and Ecology, Chongqing University, Chongqing 400044, China; huangyk2118@cqu.edu.cn (Y.H.); kousw@cqu.edu.cn (S.K.); xtzhang@cqu.edu.cn (X.Z.); wanglei137319@cqu.edu.cn (L.W.); lupl@cqu.edu.cn (P.L.)

**Keywords:** bisphenol A, peroxymonosulfate, photocatalysis, composites, Z-scheme

## Abstract

The rational fabrication of direct Z-scheme heterostructures photocatalysts is a pivotal strategy to boost the interfacial charge migration and separation. Herein, direct Z-scheme Bi_2_WO_6_/WO_3_ composites were rationally fabricated for the degradation of bisphenol A combined with the activation of peroxymonosulfate (PMS). The tight interface contact between Bi_2_WO_6_ and WO_3_ was successfully formed by the in situ epitaxial growth of ultrathin Bi_2_WO_6_ nanosheets at the surface of WO_3_ nanorods. The Bi_2_WO_6_/WO_3_ composite presented highly efficient catalytic performance toward degradation of BPA with PMS activation as compared to the WO_3_ and Bi_2_WO_6_. PMS can dramatically boost the photocatalytic activity of the composites. Moreover, the results of active radical scavenging experiments revealed that *h*^+^, •O_2_^−^, and •SO_4_^−^ are critical active species in the photodegradation reaction. Finally, the photocatalytic mechanism for the degradation of BPA is also discussed in detail. The great improvement of photocatalytic performance should be ascribed to the effective formation of the direct Z-scheme heterojunctions between Bi_2_WO_6_ and WO_3_, resulting in improved light absorption, an efficient transfer and separation of photoinduced charge carriers, and a considerable amount of the electrons and holes with strong reduction and oxidation abilities. The study might provide new inspirations to design and construct heterostructured nanomaterials with outstanding photoactivity for environmental remediation.

## 1. Introduction

Bisphenol A (BPA), as one of a well-known endocrine disrupting compounds (EDCs), has become an environmental concern [1,2,3]. BPA can be lethal to humans or cause reproductive impairment, diabetes, neurological disorders, cardiovascular disease, and carcinogenic sensitivity [1,3]. BPA is usually used as the raw material for the preparation of epoxy resins, plastics, food containers, water pipes, toys, coatings, medical equipment, and electronics [4,5]. The United States (US) Environmental Protection Agency (EPA) reports that a lot of BPA has entered to the aquatic environment, resulting the ubiquitous presence of BPA in underwater and surface water [2,3]. Thus, it is notably urgent to seek effective techniques to remove BPA from water bodies [1].

Peroxymonosulfate (PMS)-based advanced oxidation processes (AOPs) have aroused considerable attention in environmental remediation [5,6]. PMS could be activated to generate sulfate radicals (•SO_4_^−^) and hydroxyl radicals (•OH) [7,8]. The •SO_4_^−^ has been found to be a superior alternative for the environmental remediation of organic contaminants, due to its great advantage, including stronger oxidizing properties, higher oxidation selectivity, and more rapid degradation rate, in contrast to •O_2_^−^ and •OH [2,6]. PMS can be activated by multifarious methods, such as ultraviolet radiation [8], transition metal ions [9], carbon based materials [10,11], and transition metal composites [2,5]. Unfortunately, most of them are limited by the problems of an external energy input, low activation efficiency, and metal ions leaching [12]. Recently, semiconductor-based photocatalysis is manifested to be a promising strategy for PMS activation to avoid the above potential problems [8,13,14]. More importantly, the coupling of PMS activation and semiconductor-based photocatalysis can not only rapidly and effectively activate PMS toward the formation of •SO_4_^−^, but it can also efficiently accelerate the transfer and separation of photogenerated charges of photocatalysts, leading to an enhanced the catalytic performance [7,13,15]. However, the application of current photocatalytic materials in PMS activation is far from satisfactory due to its insufficient light absorption, low efficiency of photogenerated charge separation, and limited surface active sites [8,16]. Therefore, it is urgently desirable to fabricate novel photocatalytic materials that are highly active and stable for PMS activation.

Tungsten oxide (WO_3_) has been considered to be a promising photocatalyst because of its unique optical properties, low cost, chemical stability, and high photoactivity [17,18]. Unfortunately, the photoinduced electrons on the conduction band (CB) of WO_3_ cannot efficiently reduce dissolved oxygen to produce •O_2_^−^ radical due to its relatively low CB potential [17,19]. In addition, the photocatalytic applications of WO_3_ are also limited due to its inefficient light response, poor conductivity, and serious electron-hole recombination [20,21,22]. Substantial strategies have been utilized to improve its photocatalytic performances, like doping, deposition of noble metal, introducing surface oxygen vacancy, constructing rational heterostructure, etc. [17,20,23]. In particular, constructing rational heterostructure with other components, such as BiVO_4_ [21,24], g-C_3_N_4_ [22,25], and Bi_2_WO_6_ [9,26], has been reported to greatly improve the photocatalytic performances. In this regard, Bi_2_WO_6_, a classic of Aurivillius oxide, is an ideal selection for constructing heterojunction composites with WO_3_, due to its unique layered structures with composition of the layers of corner-shared WO_6_ octahedra sandwiched between the [Bi_2_O_2_]^2+^ layers [27,28]. However, a weak interactions between each component and complexity of the synthetic protocols seriously block their widespread application potential [28,29]. More unfortunately, the traditional heterojunction photocatalysts will lead to a lower reducibility and oxidizability for photogenerated electrons and holes, which are of great significance in enhancing the catalytic efficiency [22,30,31,32]. Hence, exploring the in situ formation of direct Z-scheme heterostructures would be a pivotal strategy for addressing the aforementioned problems, which not only attain the strengths of each component, but also introduce some new properties [29,33]. Nevertheless, to the best of our knowledge, the construction of the direct Z-scheme Bi_2_WO_6_/WO_3_ composite with specific structures combined with PMS activated for efficient removal of BPA has never been discovered until now.

In this work, direct Z-scheme Bi_2_WO_6_/WO_3_ composites were firstly fabricated by in situ epitaxial growth method. The Bi^3+^ ions can partially replace the water molecules at the surface of WO_3_ nanorods, resulting in a tight interface contact between ultrathin Bi_2_WO_6_ nanosheets and WO_3_ nanorods, which is critically important in constructing efficient heterojunctions. The catalytic activity of the composites were systematically assessed by the photodegradation of BPA combined with the activation of PMS. The Bi_2_WO_6_/WO_3_ composite presented highly efficient catalytic performance toward the degradation of BPA with PMS activation as compared to the WO_3_ and Bi_2_WO_6_. PMS can dramatically boost the photocatalytic activity of the composites. The possible catalytic mechanism for photodegradation of BPA was also investigated in detail. This excellent performance should be ascribed to the effective construction of the direct Z-scheme heterojunctions between Bi_2_WO_6_ and WO_3_, which facilitates the absorption capacity of visible light, promotes the efficient of charge carriers transfer and separation, and provides a considerable amount of the electrons and holes presenting strong reduction and oxidation abilities.

## 2. Materials and Experimental

### 2.1. Materials

Bismuth(III) nitrate pentahydrate (Bi(NO_3_)_3_·5H_2_O), p-benzoquinone (BZQ), BPA, and sodium tungstate dihydrate (Na_2_WO_4_·2H_2_O) were purchased from Aladdin, Shanghai, China. Hydrochloric acid (HCl), tert-butyl alcohol (C_4_H_10_O), potassium persulfate (K_2_S_2_O_8_), anhydrous ethanol, methanol, and nitric acid (HNO_3_) were purchased from Sinopharm Reagent Co., Shanghai, China.

### 2.2. Synthesis of Bi_2_WO_6_/WO_3_ Composites

The WO_3_ nanorods was fabricated via the procedure reported in previous work [34]. The Bi_2_WO_6_/WO_3_ composites were synthesized by in situ epitaxial growth method. Typically, a certain amount of Bi(NO_3_)_3_·5H_2_O was dissolved in 60 mL deionized water. 0.4 g of as-prepared WO_3_ nanorods were added in the above solution under vigorous stirring, and then sonicated for 30 min. to ensure WO_3_ nanorods disperse well. After another stirring for 30 min., the obtained solution were transferred to a 100 mL Teflon-lined autoclave, then, heated at 160 °C for 16 h. The product was separated by centrifugation, washed by anhydrous ethanol and deionized water several times, and then dried at 60 °C. The various hybrid products were obtained by changing the stoichiometric ratios (Bi:W = 0.8, 1.2, 1.4, and 1.6), which were signed as BW1, BW2, BW3, and BW4, respectively. For comparison, pure Bi_2_WO_6_ was also fabricated according to the reference [35,36].

### 2.3. Sample Characterization

The structure of the samples was characterized by the X-ray powder diffraction (XRD, PANalytical X’Pert, Osaka, Japan), Fourier transformation infrared spectra (FT-IR, Nicolet iS50 spectrometer, Thermo Fisher Scientific, Waltham, MA, USA), and Raman spectra (LabRAM HR spectroscopy, HORIBA Jobin Yvon, Longjumeau, France). The morphologies of the samples were collected with Scanning electron microscopy (JSM-7800F electron microscope, Tokyo, Japan) and field emission transmission electron microscope (TEM, Talos F200S electron microscope, Thermo Fisher Scientific, Waltham, MA, USA). The elemental mappings of the samples were carried out with an energy dispersive X-ray spectrometry (EDX, Thermo Fisher Scientific, Waltham, MA, USA). The lattice spacing were obtained by the Digital Micrograph™ software. X-ray photoelectron spectroscopy (XPS) measurement was carried out on a Thermo Fisher Escalab 250Xi X-ray photoelectron spectrometer (Thermo Fisher Scientific, Waltham, MA, USA) with Al *K*α radiation as the excitation source. The spectra of various elements were calibrated according to C 1s peak (284.8 eV), and fitted by Thermo Avantage v5.952 software with a Shirley background subtraction method. The UV-vis diffuse reflectance spectroscopy spectra (DRS, Shimadzu UV-3600 spectrophotometer with an integrating sphere attachment, Kyoto, Japan) was used to measure the optical properties. The electrochemical measurements were conducted using a standard three-electrode electrochemical system (CHI 660E electrochemical analyzer, Shanghai, China).

### 2.4. Photocatalytic Performance Tests

The photocatalytic performances of samples were implemented at about 25 °C under visible irradiation. A 300 W Xe arc lamp equipped with a glass filte (*λ* > 400 nm) (CELHXF300, China Au-light Co., Ltd., Beijing, China) was employed as the visible light source. The catalysts (50 mg) were dispersed into BPA solution (10 mg/L, 50 mL) with magnetic stirring for 30 min. in order to reach the adsorption and desorption equilibrium. After that, the reaction was initialized by adding PMS under visible light irradiation. 1 mL of aqueous sample was extracted at different intervals and 0.2 mL of methanol was rapidly added to quench the radicals. Unless otherwise specified, the solution pH was 6.9 (natural) and the amounts of PMS was 0.1 mmol/L. The BPA concentration at different time was analyzed on a liquid chromatography (HPLC, Agilent 1260) with a Agilent C-18 column and UV detection wavelength at 276 nm. The mixture solution of 30% water and 70% methanol were used as mobile phase, and the flow rate was 1.0 mL/min.

## 3. Results and Discussion

### 3.1. Characterization of Catalysts

**Figure 1:**
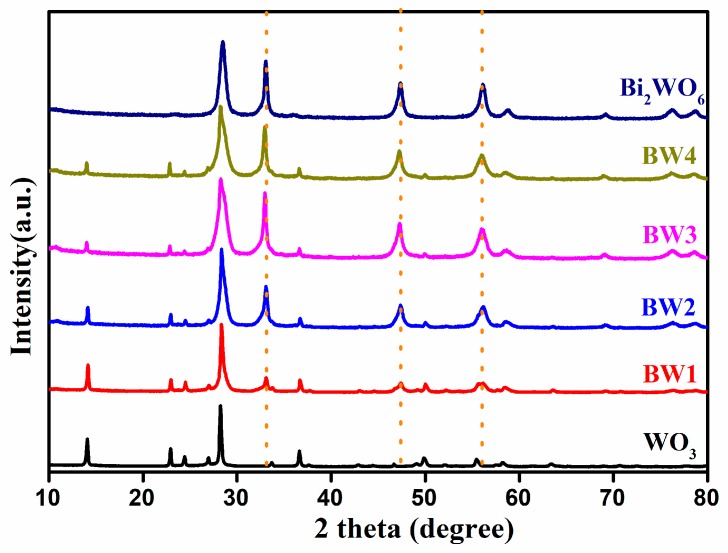
X-ray powder diffraction (XRD) patterns of pure WO_3_, Bi_2_WO_6_, and Bi_2_WO_6_/WO_3_ samples.

The crystalline structures of the prepared photocatalysts have been investigated by XRD. As shown in Figure 1, pure WO_3_ represents the main diffraction peaks at 2*θ* values of 14.1°, 22.9°, 24.4°, 27.0°, 28.3°, 36.6°, 49.2°, 50.1°, and 55.6°, which are in good agreement with the corresponding (100), (001), (110), (101), (200), (201), (301), (220), and (221) planes of hexagonal WO_3_ (JCPDS 35-1001) [23,37], respectively. For the Bi_2_WO_6_/WO_3_ composite, the new diffraction peaks at 28.3°, 32.9°, 47.1°, 55.8°, 58.5°, 76.1°, and 78.5° can be corresponded to the (131), (002), (202), (331), (262), (333), and (204) planes of orthorhombic Bi_2_WO_6_ (JCPDS 39-025), respectively [28,38]. Moreover, the signal of these peaks becomes stronger with a gradual increase of the content of Bi^3+^, which confirms the formation of Bi_2_WO_6_. In addition, no additional new phases could be observed, confirming that the high purity of the prepared samples. Notably, the Bi_2_WO_6_ are successfully grown at the surface of WO_3_ nanorods.

**Figure 2:**
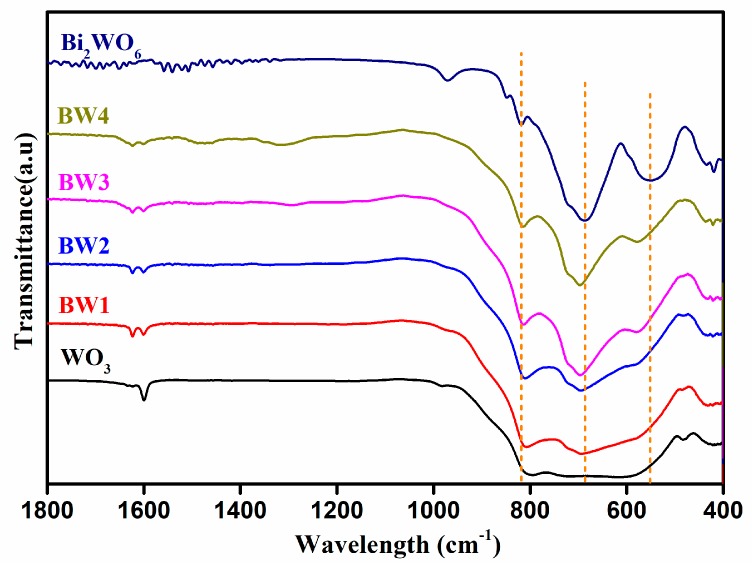
Fourier transformation infrared spectra (FT-IR) spectra of pure WO_3_, Bi_2_WO_6_, and Bi_2_WO_6_/WO_3_ samples.

The functional groups composition of Bi_2_WO_6_/WO_3_ samples were further studied by FT-IR. As shown in Figure 2, pure WO_3_ displays a broad peak at 500–900 cm^−1^, ascribable to the O–W–O stretching vibration [4,25]. The characteristic peaks of 1600 cm^−1^ is attributed to the bending vibrations of the surface hydroxyl groups, which indicates the presence of the absorbed water molecules on the surface of the sample. As for pure Bi_2_WO_6_, the peaks located at 548 and 684 cm^−1^ could be assigned to the bridging stretching modes of Bi–O, and the bands of about 820 cm^−1^ belong to the sharing stretching modes of W–O–W [39]. As for the Bi_2_WO_6_/WO_3_ samples, the typical peaks of WO_3_ and Bi_2_WO_6_ are found in the as-prepared Bi_2_WO_6_/WO_3_ composite. With compositional proportion of Bi in the composites increasing, the peak intensities of the Bi_2_WO_6_ increase, while those of the WO_3_ lower gradually. Moreover, the characteristic peaks of Bi_2_WO_6_ have a clear peak shift, manifesting the good integration in Bi_2_WO_6_/WO_3_ composite is well constructed [4,28]. These results further prove the successful combination of WO_3_ nanorods and Bi_2_WO_6_ in the composites.

**Figure 3:**
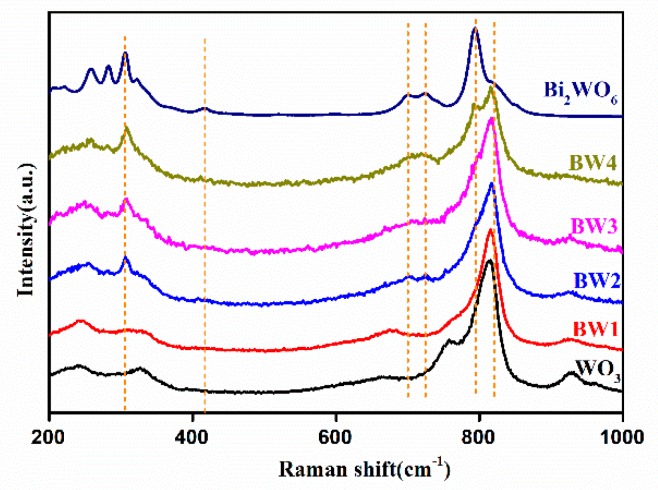
Raman spectra of pure WO_3_, Bi_2_WO_6_, and Bi_2_WO_6_/WO_3_ samples.

Raman spectroscopy is used to characterize the bonding states and the local structure of composites. In the Raman spectrum of the pure WO_3_ (Figure 3), the band located at 245 and 328 cm^−1^ can be assigned to the O–W–O bending vibration mode, while those band located at 756 and 815 cm^−1^ are ascribed to the W–O stretching vibration mode [4]. For the pure Bi_2_WO_6_, the band at 309 cm^−1^ is ascribed to the translational modes of Bi^3+^ and WO_6_^6−^, the band at 417 cm^−1^ is associated with the WO_6_ bending (Eu) modes, the band at 700 and 725 cm^−1^ reflect the asymmetric stretching vibration of W–O bond, and the bands at 793 and 820 cm^−1^ are assigned to the terminal O–W–O antisymmetric and symmetric Ag modes [27,28,40]. Bi_2_WO_6_ and WO_3_ are both detected in the composites. These characteristic peaks at 245 and 328 cm^−1^ gradually decreased with the content of Bi_2_WO_6_ increased, which demonstrates that the contact between WO_3_ and Bi_2_WO_6_ affect the O–W–O bending vibration mode of WO_3_ [28,40]. These results further signify the tight contact between Bi_2_WO_6_ and WO_3_ in the Bi_2_WO_6_/WO_3_ composites.

**Figure 4:**
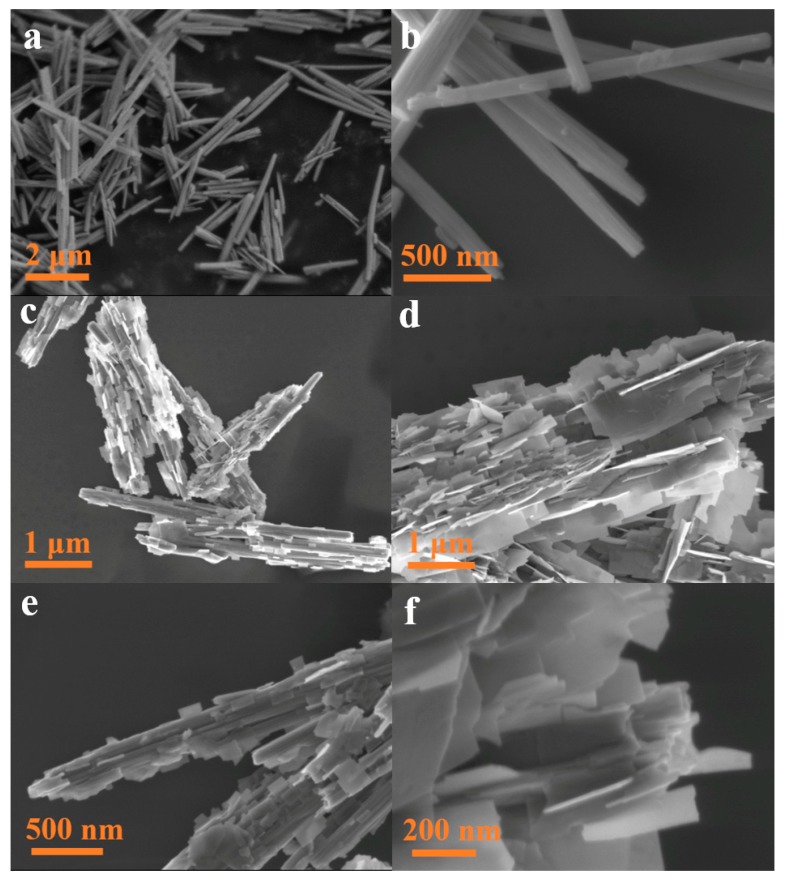
The SEM images of WO_3_ (**a**,**b**) and BW3 (**c**–**f**).

SEM was employed to investigate the microstructure of the obtained samples. As presented in Figure 4a,b, the synthesized WO_3_ displayed the rodlike morphology, with the diameter from 50 to 200 nm and length from 1 to 3 μm. Figure 4c–e illustrate that the nanocomposites has a multi-slice structure, in which the WO_3_ NRs are completely enveloped by the ultrathin Bi_2_WO_6_ nanosheets. These nanosheets are grown at the surface of WO_3_ nanorods and are oriented to different angles. The high-resolution SEM (Figure 4f) indicate these nanosheets have a rough surface and uniform thickness of approximately 8 nm and the length ranged from 200 nm to 2 μm, which could bring more coordinatively nonsaturable surface-active sites for catalytic reaction. The above results prove that an intimate contact between the Bi_2_WO_6_ nanosheets and WO_3_ nanorods can be readily fabricated by the in situ epitaxial growth method.

**Figure 5:**
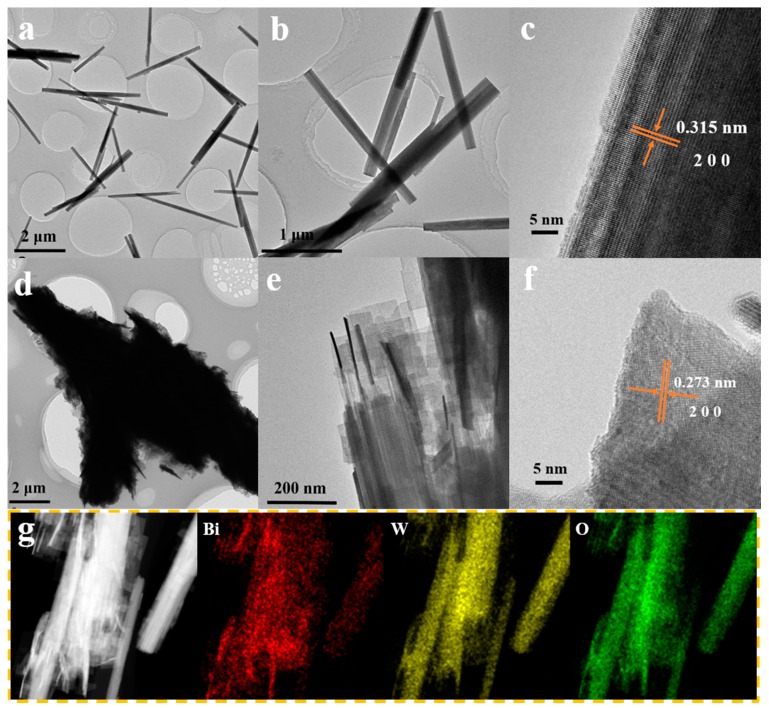
The transmission electron microscope (TEM) and high-resolution-TEM (HRTEM) image of WO_3_ (**a**–**c**) and BW3 (**d**–**f**) and the STEM mapping (**g**) of BW3.

The microstructure of the samples were further analyzed by TEM and HRTEM, and the results are presented in Figure 5. WO_3_ shows the rod shaped structure with the diameter from 50 to 200 nm, length from 1 to 3 μm. As shown in Figure 5c, the interplanar distance of 0.315 nm is clearly observed, which corresponds to the (200) plane of hexagonal WO_3_. Figure 5d,e reveals the BW3 sample was assembled by the ultrathin nanosheets, which are grown from the surface of WO_3_ nanorods, and oriented to different angles. The single nanosheet is very thin and estimated to be less than 10 nm. The HRTEM image (Figure 5f) of BW3 sample reveals that these nanosheets exhibit highly crystalline. Furthermore, the lattice distance of 0.273 nm can be clearly identified which corresponds to (200) planes of Bi_2_WO_6_ [40,41]. In addition, EDS mappings (Figure 5g) reveal that the Bi, W, and O elements are homogeneously distributed over the entire sample. These observations confirm the effective formation of heterojunctions between Bi_2_WO_6_ and WO_3_, which is beneficial for the improvement of photocatalytic efficiency [30,33]. These results are in good agreement with the results of the XRD, Raman, and SEM.

**Figure 6:**
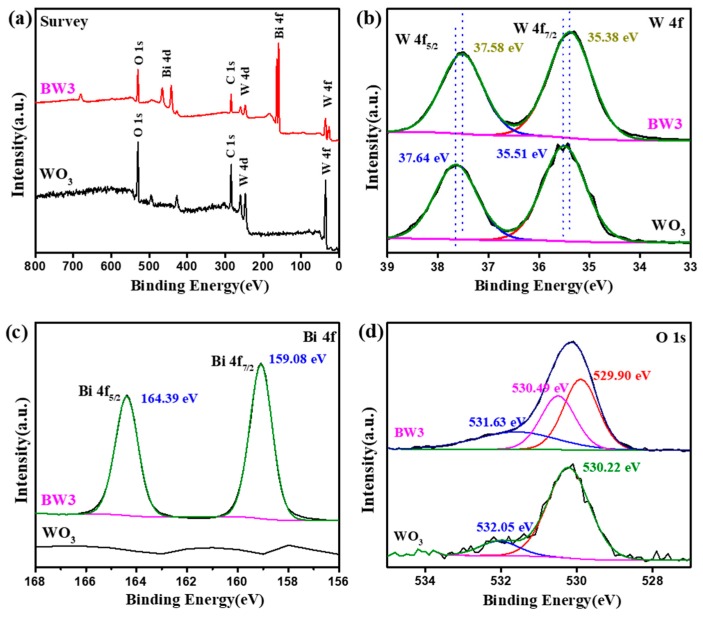
The X-ray photoelectron spectroscopy (XPS) spectra of WO_3_ and BW3. (**a**) survey spectra, (**b**) W4f, (**c**) Bi4f, and (**d**) O1s.

The surface chemical states of WO_3_ and BW3 were detected by XPS [42]. The survey spectra of WO_3_ and BW3 composite are presented in Figure 6a. It can be observed that the WO_3_ contains W and O elements, while the BW3 composite contain Bi, W, and O elements, validating Bi_2_WO_6_ exist in the BW3 composite. The high-resolution spectrum of W 4f (Figure 6b) exhibits two peaks at binding energy of 35.51 and 37.64 eV, which are assigned to W 4f_7/2_ and W 4f_5/2_ of W^6+^ in WO_3_ [19,22]. The characteristic peak of the W 4f in the BW3 composite have slightly shifted to 37.58 and 35.38 eV, which can be assigned to W 4f_5/2_ and 4f_7/2_, respectively, which indicated that W^6+^ presents in BW3 [43]. From the high-resolution spectrum of Bi 4f (Figure 6c), two characteristic peaks at 164.39 and 159.08 eV are observed in the spectra of BW3, which can be assigned to Bi 4f_5/2_ and Bi 4f_7/2_ from Bi^3+^ in Bi_2_WO_6_, respectively [28,43]. For pure WO_3_, the O 1s XPS spectra (Figure 6d) centered at 529.6 eV is deconvoluted to characteristic peaks at 530.22 and 532.05 eV, assigned to oxygen group from lattice oxygen and adsorbed oxygen species, respectively [20,30,34]. Nevertheless, the O 1s spectrum of BW3 could be deconvoluted to three peaks at 529.90, 530.49, and 531.63 eV, which are derived from lattice oxygen of [Bi_2_O_2_]^2+^ and [WO_4_]^2−^ and the adsorbed hydrated species, respectively [15,40]. The results demonstrate that the surfaces of WO_3_ nanorods are well covered by Bi_2_WO_6_. These findings further demonstrate that the Bi_2_WO_6_ are successfully in situ grown at the surface of WO_3_ nanorods.

**Figure 7:**
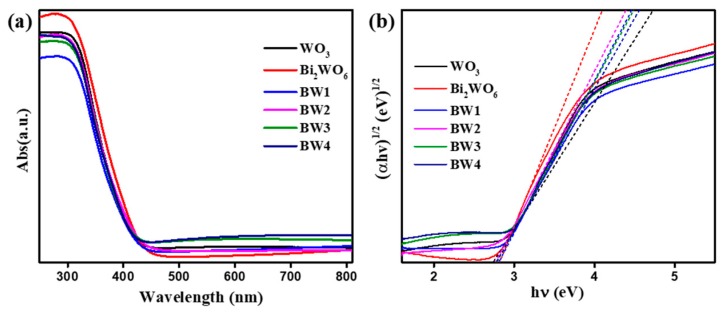
UV-vis diffused reflection spectra (**a**) and Tauc’s plots (**b**) of pure WO_3_, Bi_2_WO_6_, and Bi_2_WO_6_/WO_3_ samples.

The photoabsorption properties of the prepared samples were studied by UV-vis diffuse reflectance spectra (DRS). As shown in Figure 7a, the WO_3_ exhibits a absorption edge appeared near 470 nm due to its intrinsic bandgap [20,30]. The pure Bi_2_WO_6_ displays a absorbance edge at ca. 450 nm because of the charge transfer response of Bi_2_WO_6_ [36,44]. Obviously, the light absorption intensity of the composites gradually improved with increasing Bi_2_WO_6_ loadings. It is noted that the absorption edge of the composite has a slight red shift, while the absorption ability of the composite samples increased remarkably. Moreover, the band gap of the samples are acquired according to Kubelka-Munk method (Figure 7b). The band gap of WO_3_, Bi_2_WO_6_, BW1, BW2, BW3, and BW4 are estimated to be 2.74, 2.81, 2.80, 2.78, 2.77, and 2.75 eV, respectively. This result unambiguously confirms the formation of Bi_2_WO_6_/WO_3_ heterojunction structure. Therefore, it is reasonable to infer that the composite samples could harness more visible light, and form more electron-hole pairs, signifying the superior photocatalytic activity.

### 3.2. Photocatalytic Performance

**Figure 8:**
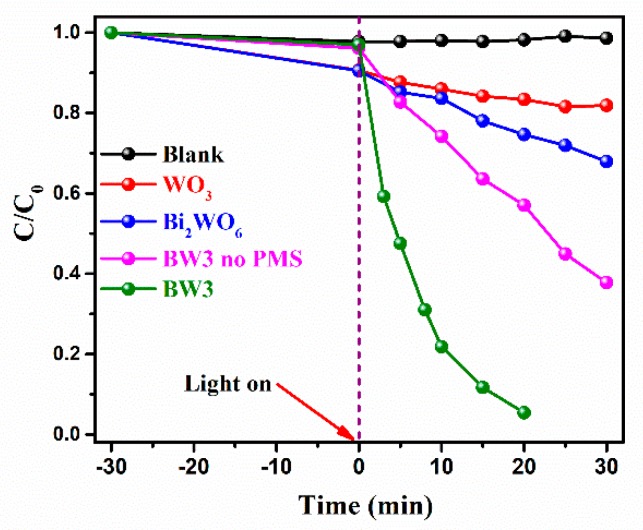
The degradation efficiency of Bisphenol A (BPA) under visible light irradiation over various samples. Reaction condition: initial BPA concentration of 10 mg/L, peroxymonosulfate (PMS) dosage of 0.1 mmol/L, catalyst addition of 1 g/L.

The photodegradation of BPA in the presence of PMS was employed as the probe reaction to evaluate the photocatalytic efficiency of the prepared materials. As depicted in Figure 8, the prepared materials have different adsorption capacity. It can be seen that 9.5%, 9.4%, and 3.9% of BPA were adsorbed by WO_3_, Bi_2_WO_6_, and BW3 within 30 min., respectively. Obviously, the prepared materials show only small amounts of BPA adsorption abilities. Under visible-light irradiation, the remove efficiency is very low in the absence of photocatalysts, indicating that single PMS could hardly degrade BPA [7,13,14]. Moreover, WO_3_ and Bi_2_WO_6_ exhibit weak photocatalytic performance with PMS activation, and only 18.1 and 32.0% of BPA are removed within 30 min., respectively. Clearly, the BW3 exhibits remarkable photocatalytic efficiency as compared to the WO_3_ and Bi_2_WO_6_. Only 43.0% of BPA can be degraded by BW3 after 20 min. of visible light irradiation, while removal efficiency can be achieved to 94.7% with PMS activation. These results unambiguously proves that the introduction of PMS effectively improves the photocatalytic efficiency of the composites. Therefore, it is concluded that the heterojunction is formed between the interfaces of Bi_2_WO_6_ and WO_3_, thus leading to promoting the photocatalytic activity in PMS activation and photodegradation of BPA.

**Figure 9:**
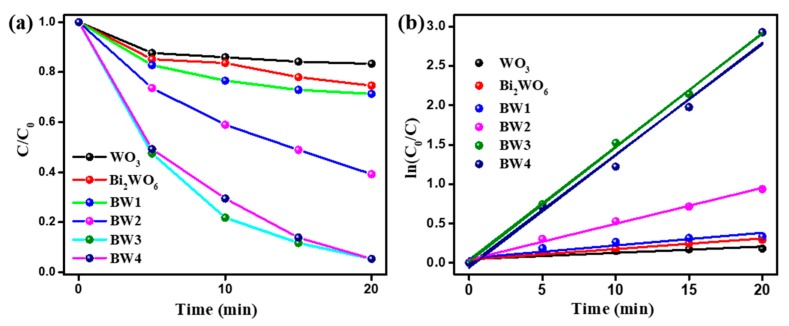
The influence of stoichiometric ratios of Bi and W in the composites for the BPA degradation (**a**), and the corresponding degradation kinetics of BPA (**b**). Reaction condition: initial BPA concentration of 10 mg/L, PMS dosage of 0.1 mmol/L, catalyst addition of 1 g/L.

The influence of stoichiometric ratios of Bi and W in the composites for the BPA degradation was further evaluated. As illustrated in Figure 9a, the stoichiometric ratios of Bi and W has a significant influence on photodegradation of BPA with PMS activation. As the stoichiometric ratios of Bi and W increased from 0.8 to 1.4, the photocatalytic activities of the composites improve gradually and the BW3 shows the highest photocatalytic activity. It is mainly attributed to the effective formation of heterojunctions between two components, which could enhance the visible light absorption and improve the separation efficiency of charge carriers in the composites. However, the catalytic efficiency decreased with further increasing the stoichiometric ratios of Bi:W to 1.6. It is concluded that excessive Bi_2_WO_6_ at the surface of the WO_3_ would dramatically decrease active sites on the heterojunctions. Furthermore, the photocatalytic degradation kinetics of BPA can be further evaluated by fitting the experimental data to following pseudo first-order kinetics Equation (Figure 9b). The apparent rate constant (*k*) is estimated to be 0.008, 0.015, 0.016, 0.045, 0.145, and 0.143 for WO_3_, Bi_2_WO_6_, BW1, BW2, BW3, and BW4, respectively. The BW3 sample exhibits the highest photodegradation rate of BPA with PMS activation, which is approximately 18.1 and 9.6 times higher than that of WO_3_ and Bi_2_WO_6_. Therefore, suitable stoichiometric ratios of Bi:W in the composites are necessary for optimizing the catalytic activity for BPA photodegradation with PMS activation.

### 3.3. Photodegradation Mechanism of the Samples

**Figure 10:**
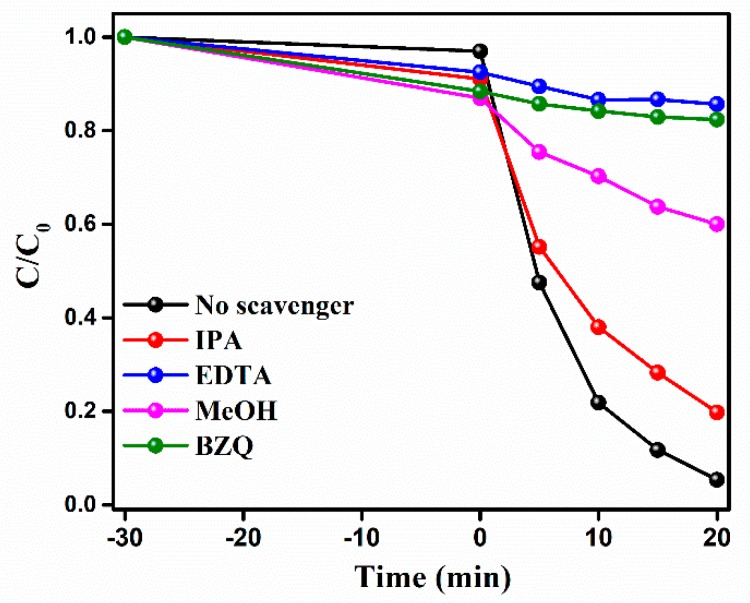
The photocatalytic degradation of BPA over BW3 with PMS activation in the presence of different scavengers. Reaction condition: initial BPA concentration of 10 mg/L, PMS dosage of 0.1 mmol/L, catalyst addition of 1 g/L.

The active radicals that were involved in the photocatalytic degradation of BPA under the BW3 with PMS activation were investigated by the radical trapping experiments. In these process, isopropanol (IPA, 10 mmol/L), ethylenediaminetetraacetic acid disodium salt (EDTA, 10 mmol/L), methanol (MeOH, 10 mmol/L), and benzoquinone (BZQ, 0.1 mmol/L) were severed as the scavengers for hydroxyl radical (•OH), holes (*h*^+^), and sulfate radical (•SO_4_^−^) and superoxide radical (•O_2_^−^), respectively [7,13]. Figure 10 shows that the photocatalytic activity slightly decreased in the presence of IPA, which implied that the •OH has a little role in photodegradation reaction. Moreover, the introducing BZQ, EDTA, and MeOH in the reactive solutions can lead to a significant inhibition for BPA degradation. Overall, the inhibition for BPA degradation ranked in the sequence of EDTA > BZQ > MeOH > IPA. The above results exhibit that *h*^+^, •O_2_^−^, and •SO_4_^−^ are major active species for BPA degradation over BW3, while the •OH plays a minor role in reaction process.

**Figure 11:**
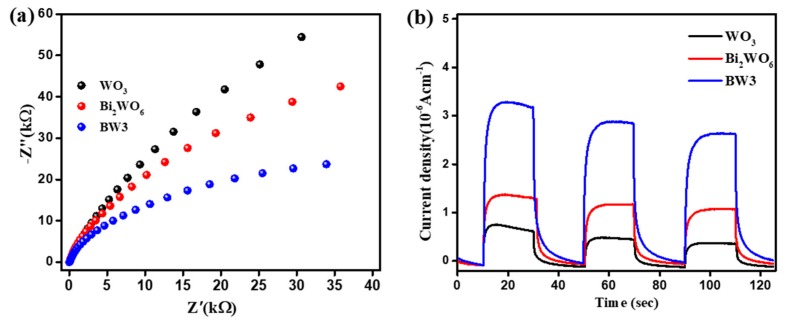
Electrochemical impedance spectra (EIS) (**a**) and photocurrents responses (**b**) of WO_3_, Bi_2_WO_6_ and BW3 composite.

The electrochemical impedance spectra (EIS) and photocurrent responses were conducted to explore the interfacial charge separation efficiency, and the results are depicted in Figure 11. Generally, a lower radius of the Nyquist plot represents a faster interfacial charge transport [45,46]. As shown in Figure 11a, the arc radius of EIS Nyquist circle of BW3 is smaller than those of single WO_3_ and Bi_2_WO_6_. The results reveal that BW3 exhibits highly efficient separation and transfer of photoinduced charge carriers. Moreover, the photocurrent density of 0.7, 1.4 and 3.3 μAcm^−2^ can be obtained for WO_3_, Bi_2_WO_6_ and BW3 under identical conditions, respectively (Figure 11b). Obviously, BW3 has superior photocurrent response. Therefore, it can be inferred that the photoinduced charge carriers in the Bi_2_WO_6_/WO_3_ composites can fleetly transfer between the WO_3_ and Bi_2_WO_6_, and the formed heterojunction interface effectively inhibited the direct recombination of electron-hole pairs.

**Figure 12:**
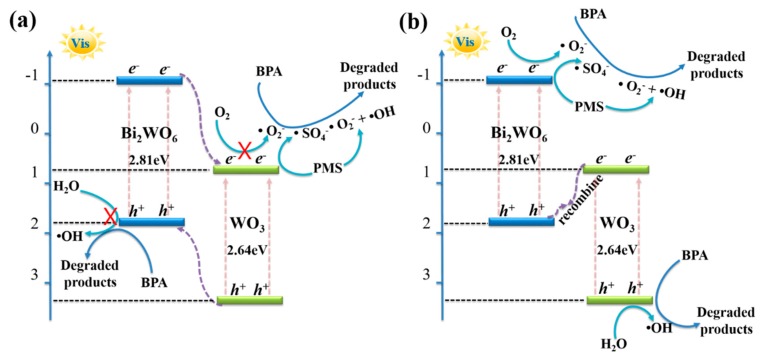
The proposed catalytic mechanisms of Bi_2_WO_6_/WO_3_ composite: (**a**) the type-II heterojunction; (**b**) the Z-scheme.

Based on these analyses, the possible catalytic mechanism for BPA photodegradation over the Bi_2_WO_6_/WO_3_ composites with PMS activation can be schematically illustrated in Figure 12. The WO_3_ and Bi_2_WO_6_ could generate the photogenerated holes and electrons under visible light irradiation, because their band gaps are about 2.64 and 2.81 eV, respectively [22,39]. In addition, the CB and VB edge position of Bi_2_WO_6_ is −1.04 and 1.77 eV (vs NHE), and the CB and VB edge position of WO_3_ is 0.77 and 3.41 eV (vs NHE), respectively [22,47,48]. Therefore, two kinds of transfer and separation of carrier transfer might be possible, according to the band arrangement principle. The first type is the type-II heterojunction architectures (Figure 12a). When irradiated by visible light, WO_3_ and Bi_2_WO_6_ are both excited to generate electrons-holes pairs. Subsequently, the generated electrons of CB in Bi_2_WO_6_ can inject into the CB of WO_3_, and the holes could cross from the VB of WO_3_ to the VB of BW. Hence, the electrons are accumulated in the CB of WO_3_, and the holes are accumulated in the VB of Bi_2_WO_6_. However, the photogenerated electrons could not efficiently react with dissolved oxygen to generate •O_2_^−^ radicals, because the position of CB of WO_3_ is lower than the standard potential of O_2_/•O_2_^−^ (−0.33 eV vs. NHE) [22,37]. Meanwhile, the holes on the VB of Bi_2_WO_6_ are too weak to generate •OH [39,47]. Therefore, a heterojunction-type electron transfer is not appropriate in the present system, which is inconsistent with the results of the trapping radical experiment.

Therefore, the Z-scheme mechanism would properly explain the photocatalytic process and is shown in Figure 12b. In this case, the photogenerated electrons are transferred from the CB of WO_3_ to the VB of Bi_2_WO_6_, and recombined with the photogenerated holes that are generated on the VB of Bi_2_WO_6_, resulting in abundant photoexcited electrons on the CB of the Bi_2_WO_6_, and holes on the VB of WO_3_. Consequently, these photogenerated charge carriers have supreme oxidation and reduction abilities to stimulate the photocatalytic process. The electrons accumulated in the CB of the Bi_2_WO_6_ can react with dissolved oxygen to generate •O_2_^−^ radicals. Simultaneously, the electrons could be efficiently captured by PMS to generate •SO_4_^−^, which can not only provide highly reactive oxygen species for BPA photodegradation, but also greatly accelerate the separation of charge carriers [13,14]. The photoexcited holes that accumulated on the VB of WO_3_ can directly oxidize BPA into the nontoxic products. The •OH are produced by the oxidize reaction of the holes with surface-adsorbed water or OH^-^ on the surfaces of BW. Besides, •OH can also be generated by the conversion of •O_2_^−^ or PMS [7,13]. Consequently, the •SO_4_^−^, •O_2_^−^, *h*^+^, and •OH can efficiently decompose BPA in the photocatalytic process. Therefore, it can be concluded that the effective formation of Z-scheme heterojunctions between Bi_2_WO_6_ and WO_3_ facilitates the absorption capacity of visible-light, promotes the efficient transfer and separation of charge carriers, and provides more supreme oxidation and reduction abilities of electrons and holes.

## 4. Conclusions

In summary, direct Z-scheme Bi_2_WO_6_/WO_3_ composite was rationally fabricated in order to degrade BPA with the activation of PMS under visible light irradiation. The tight interface contact between Bi_2_WO_6_ and WO_3_ is successfully realized by in situ epitaxial growth of ultrathin Bi_2_WO_6_ nanosheets at the surface of WO_3_ nanorods. The Bi_2_WO_6_/WO_3_ composites exhibited remarkably enhanced photocatalytic activity toward the degradation of BPA with PMS activation as compared to the WO_3_ and Bi_2_WO_6_. PMS could dramatically boost the photocatalytic activity of the composites. In addition, the stoichiometric ratios of Bi:W has significant influence on the photocatalytic activity of the composites. Moreover, the results of trapping radical experiment reveals that *h*^+^, •O_2_^−^, and •SO_4_^−^ are critical active species in the photocatalytic reaction. Finally, the possible catalytic mechanism for BPA degradation was also discussed in detail. Such an excellent performance should be attributed to the effective formation of direct Z-scheme heterojunctions between Bi_2_WO_6_ and WO_3_, which facilitates the capacity of light absorption, promotes the efficient transfer and separation of photoinduced charge carriers, and provides more supreme oxidation and reduction abilities of the electrons and holes. The study might provide new inspirations to design and construct heterostructured nanomaterials with outstanding photoactivity for environmental remediation.
